# A School Eye Health Rapid Assessment (SEHRA) planning tool: Module to survey the magnitude and nature of local needs

**DOI:** 10.1186/s12889-022-13927-x

**Published:** 2022-09-02

**Authors:** Priya Morjaria, Jessica Massie, Andrew Bastawrous, SEHRA Author Group, Haroon Awan, Rishi Raj Borah, Anne Buglass, Nathan Congdon, Amanda Davis, Sarity Dodson, Hannah Faal, Clare Gilbert, May Ho, Drew Keys, Hans Limburg, Islay MacTaggart, Ian McCormick, Kovin Naidoo, Naomi Nsubunga, Heiko Philippin, Mansur Rabiu, Jacqui Ramke, Serge Resnikoff, Yuddha Sapkota, B. R. Shamana, Elizabeth Smith, Jude Stern, Beatrice Varga, Sumrana Yasmin

**Affiliations:** 1 International Centre for Eye Health, Clinical Research Department, London School of Hygiene and Tropical Medicine, London, UK; 2 Peek Vision, London, UK

**Keywords:** Eye conditions, Vision impairment, Eye health, Methodology, Developing countries, Child and adolescent health

## Abstract

**Background:**

Eye conditions in children can have negative consequences on visual functioning and quality of life. There is a lack of data on the magnitude of children with eye conditions who need services for effective planning of school eye health programmes. To address this, the School Eye Health Rapid Assessment (SEHRA) tool is being developed to collect data to support school eye health programme planning.

**Methods:**

The module, ‘the magnitude and nature of local needs in school children’ is the first of six modules in the SEHRA tool. The module outlines a school-based cluster survey designed to determine the magnitude of eye health needs in children. This paper outlines the survey sampling strategy, and sample size calculations.

**Results:**

The requirements for the SEHRA survey indicate that in regions where a larger sample size is required, or where fewer schools are recruited to the survey, confidence in the accuracy of the data will be lower.

**Conclusions:**

The SEHRA survey module ‘the magnitude and nature of local needs in school children’ can be applied in any context. In certain circumstances, the confidence in the survey data will be reduced.

**Supplementary Information:**

The online version contains supplementary material available at 10.1186/s12889-022-13927-x.

## Introduction

Vision impairment (VI) in children can have long-lasting consequences on visual functioning [1], behavioural development [2] and quality of life [3], with potential consequences for future economic status [4]. Importantly, for school-going children, VI can also reduce academic achievement which contributes to low self-esteem and confidence [5]. Globally, 70.2 million children aged 0–14 years are vision impaired or blind [6], and uncorrected refractive errors (URE) are the leading cause [7–9]. The main types of refractive error are myopia, hyperopia and astigmatism.

Myopia has been increasing globally, and East Asia is the most affected region, where the prevalence doubled between 1987 and 2010 [10], and where up to 90% of high school graduates have myopia [11]. High degrees of myopia (≤ − 6.0D), increases the risk of sight-threatening conditions later in life such as myopic maculopathy, chorioretinal changes, glaucoma, cataract and retinal detachment [12, 13]. The prevalence of hyperopia is generally higher than myopia in preschool-age children (< 5 years of age), but this reverses as children become older when myopia predominates [14]. Children with hyperopia are also at a greater risk of more difficult-to-manage conditions such as strabismus and amblyopia [15–17]. Increasing hyperopia of ≥ 3.0D in children impacts visual function including a reduction in near visual acuity, near stereoacuity, accommodative response, and significantly reduced performance in literacy testing [18–20]. Early detection of astigmatism in children is also important given the increased risk of amblyopia and myopia [21–23].

Children may also have conditions which usually do not lead to VI (non-vision impairing conditions). Common non-vision impairing eye conditions include chronic allergic eye disease [24–27], infectious conjunctivitis [28, 29], blepharitis [24, 30–32], chalazion [31–33], and strabismus without amblyopia [24, 34]. These conditions are often chronic and are associated with reduced health-related quality of life of both the child and their parents [35], and often require long-term care and treatment provided by a trained eye health worker [36].

Globally, more children and adolescents are enrolled in pre-primary, primary and secondary schools than ever before, despite the population aged 0–15 years being relatively stable over the last 50 years. On any given school day prior to the COVID-19 pandemic, one billion children were in attendance [37]. However, school enrollment and attendance do not necessarily translate into effective learning, as good vision is an important requirement for effective learning [38, 39]. One approach to mitigating the potential impact of VI on learning is to deliver vision and eye health services through school-based eye health (SEH) programmes.

Historically, surveys which have estimated the prevalence of VI in school children have focused on myopia and astigmatism, which can be readily detected by measuring distance visual acuity, resulting in fewer data on hyperopia where distance visual acuity can be normal. There is also a paucity of prevalence data on non-vision impairing eye conditions; reporting these eye conditions is essential for planning, monitoring and evaluation. Another limitation of the available data is that standard definitions have not been used, and the studies are resource and time-intensive [8].

How SEH programmes are delivered and monitored are also not standardized. The visual acuity level at which children are classified as screening failures, and whether one or both eyes are affected, are also not standardized. In addition, non-vision impairing eye conditions are not routinely included in SEH programmes, nor is the eye health of teachers. Lastly, compliance with spectacle wear is not routinely monitored, and when it has been assessed was often low [40, 41].

These gaps highlight the need for low-cost, rapid approaches to generating service delivery data which are embedded within SEH programmes, to monitor screening failure, referral attendance, and compliance with treatment or spectacle wear. Ideally, data systems should enable problems to be identified and addressed as they occur, to improve efficiency.

To address the gaps and inconsistencies outlined above, the School Eye Health Rapid Assessment tool (SEHRA) is being developed to support program planners, service providers, and funders to inform the planning, implementation and monitoring of a SEH program. The rationale for a SEHRA tool is to maximise the effective allocation of scarce resources and improve the standardisation and effectiveness of SEH programs. The tool will have six core modules: 1 Assessing the magnitude and nature of local needs, 2. Human resources, 3. Referral pathways, 4. Spectacle and medication supply chains, 5. Barriers and challenges to receiving and adhering with spectacle wear and other treatments, and 6. Cross-cutting/other priorities.

The purpose of SEHRA module is to provide a standardised, rapid survey methodology to determine the eye health needs of school children to support planning and monitoring SEH programmes. In this paper, the rationale for the SEHRA sample size calculator for this module are presented.

## Methods

**Fig. 1:**
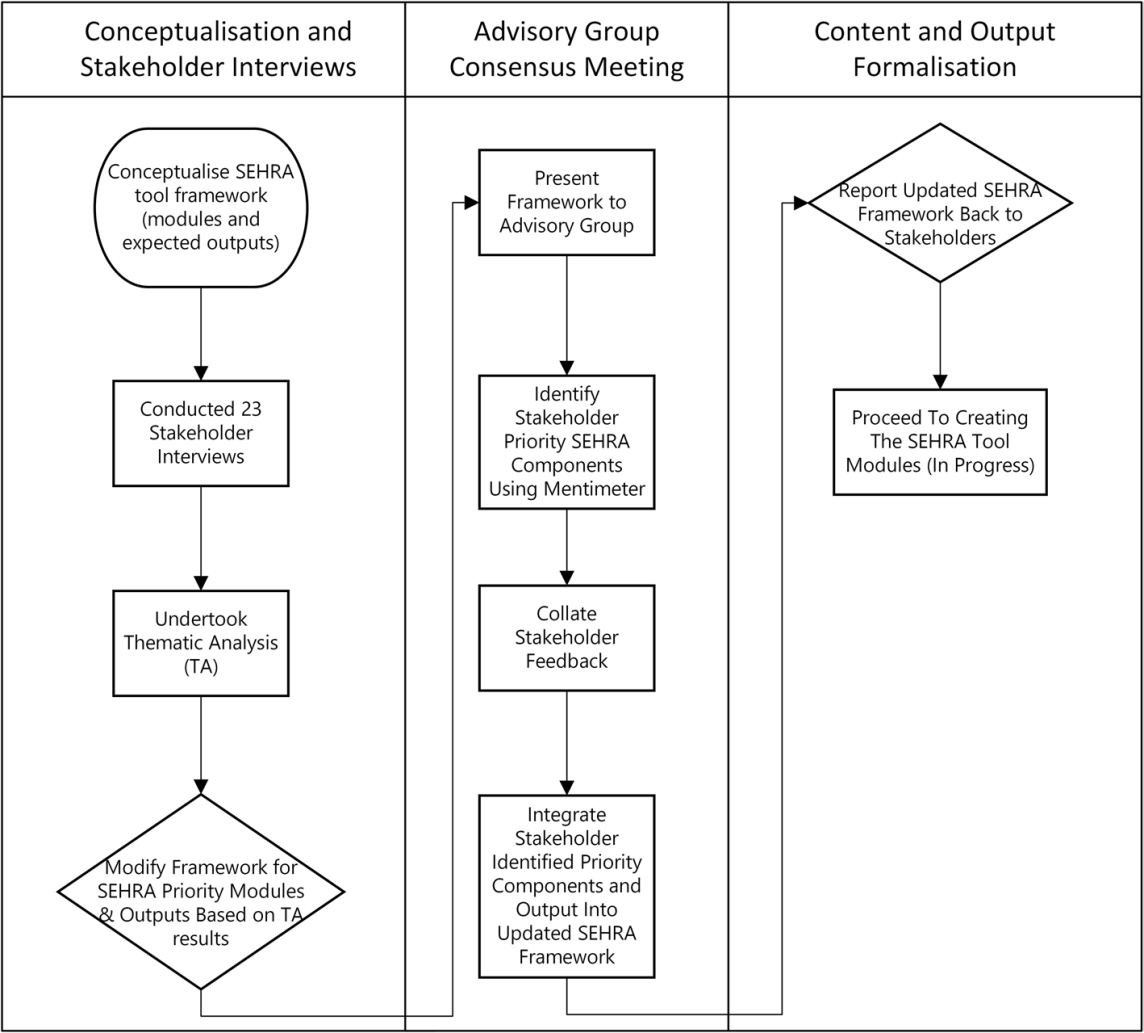
SEHRA framework and formalisation key steps

The methods for the design of the SEHRA module ‘Assessing the magnitude and nature of local needs’ involved a number of key steps (detailed in Fig. 1). Expert consultation with the SEHRA advisory group was undertaken to determine the core modules for the SEHRA and essential requirements. The advisory group was comprised of 30 members, including key user groups: implementers, funders, researchers, and persons working in advocacy, strategy, and policy. This consultation included individual semi-structured interviews, and an advisory group meeting, this process informed the topics to be taken into consideration when designing the survey. The process included a thematic analysis of individual interviews [42], and identifying priority areas and content captured via the use of the Mentimeter tool during the advisory group meeting (detailed in a forthcoming publication). A scoping literature search was subsequently conducted in July 2021, and included a review of all publications in Google Scholar reporting the prevalence of eye conditions in school children, using the search terms: “school children” AND “vision impairment” OR “non-vision impairing eye conditions”. All results were screened; the inclusion criteria included: 1) school-age children (6–17 years), 2) the publication reports vision impairment and/or NVICs. Studies identified were mapped to the relevant Global Burden of Diseases (GBD) super region and subsequently ordered according the highest and lowest prevalence in each super region. GBD super region was the chosen grouping as it is based on both gross domestic product and geographical proximity to produce the seven regional groupings [43].

This paper details a number of SEHRA sample size calculation models, demonstrating the impact of modifying various sample size calculation inputs. These models detailed below, were then undertaken to inform the design of the cluster survey, and to determine the feasibility of a SEHRA survey in the different GBD super regions. Pragmatic choices were then made regarding the design of the cluster survey and the activities required to deliver the survey.

The SEHRA tool is designed for use in all resource settings. This module: ‘Assessing the magnitude and nature of local needs’ will determine the number of school children within a defined region with eye conditions which require eye care services. This module requires a cluster survey to identify the magnitude of school children requiring services. The design of the cluster survey, including the sample size calculator, required multiple steps outlined below. The time required to conduct the SEHRA survey will vary depending on the prevalence of eye conditions in the region as this determines the sample size.

### Step 1: Summarise the prevalence of VI in GBD regions

In order to estimate the highest and lowest number of school children with eye conditions needing services across all GBD regions, a sample size calculation is required. The first step in designing the sample size calculator was to identify the highest and lowest prevalence estimates of VI in school children in each GBD region. These data were identified through the scoping literature search outlined above (see Supplementary Information, Additional file 1).

### Step 2: Estimating the proportion of children with eye conditions requiring eye care services

Data from studies by He et al. [44] and Rono et al. [45] were used for the following reasons: across GBD regions, the two studies were chosen to represent the highest and lowest prevalence of children with eye conditions requiring eye care services, as both studies reported data on VI and non-impairing conditions. The studies do not represent the absolute maximum and minimum reported prevalence of children with eye conditions requiring eye care services across GBD regions. The causes of VI included URE and other conditions such as cataract. Numerators for VI due to URE and other conditions causing VI were reported separately in the He et al. paper, and data on non-vision impairing eye conditions requiring services were extracted manually (by PM and JM). The two numbers were combined to give a total numerator from which the prevalence of conditions requiring services was calculated. Rono et al. did not report VI due to other conditions or non-vision impairing eye conditions separately; in determining the numerator for VI due to other conditions, non-vision impairing data from the He et al. publication was used to support the calculation of the estimate.

### Step 3: Cluster survey design

The SEHRA survey has a two-stage random cluster survey design. This approach has been used in other rapid eye health surveys [46], and is preferable to simple random sampling as it is not necessary to enumerate all school children in the population in advance. Schools are the primary sampling units, selected using probability proportionate to their size (number of pupils enrolled). A sampling frame of eligible schools can be obtained from the Ministry of Education or other relevant authority. Within selected schools, a set number of children will be selected at the second stage of sampling which means that every child enrolled in school in the sampling area will have an equal chance of selection. Stratification by age groups will not be included as the survey is designed to collect planning data for schools; a sub-group analysis by age can occur following data collection, if required. No stratification is required at the primary sampling unit level; however, where feasible an equal number of children will be selected across year groups.

### Step 4: Estimation of a design effect

It is necessary to apply a design effect (DEFF) to the sample size to account for the clustered nature of the data collected. This correction factor is required to allow for a loss of variation in the sample. A sensitivity analysis was undertaken to model the anticipated effect of increasing the design effect on the sample size. The true design effect could be derived from extensive pilot surveys in the area of interest, or a review of the literature of similar surveys.

### Step 5: Sample size calculations for different GBD super regions

Sample sizes for each region were calculated to demonstrate the sample sizes required to detect two groups of children who need services. That is, 1) the number of children that have an eye condition requiring referral (VI and non-vision impairing eye conditions), and 2) the number of children that have VI (all causes). The threshold of < 6/12 was selected as the World Health Organisation (WHO) defines this level as an unmet need [47], and most school surveys apply this visual acuity threshold. The following formula was used for the sample size calculation:

$$n=\frac{({Z}^{2})P(1-P)}{{d}^{2}}$$

where $n=$ the sample size required; *p* = the prevalence of children with an eye condition; ${Z}^{2}$ $=$ multiplier for alpha of 0.05; ${d}^{2}=$ desired precision, taken in relative terms as ± 20% of the prevalence.

Sample size calculations were based on the estimated prevalence range (3.9–18.9%) of school children across GBD regions requiring services (Table 2). Other parameters were as follows: 80% power, 95% confidence interval, a design effect of 2.0 and a precision of ± 20% of the prevalence. In line with the published literature, a 20% non-participation and absenteeism rate was selected [44, 48–50].

## Results

### Step 1: Summarise the prevalence of VI in GBD regions

South-East Asia, East Asia & Oceania, and Sub-Saharan Africa were identified in the scoping literature review as regions with the highest and lowest estimates for each GBD region, respectively (Table 1). Sample size calculations modelled in the remainder of this paper used data from He et al. [44], and Rono et al. [45] which best represent the highest and lowest prevalence of VI across GBD regions.

**Table 1:** Vision impairment in school-age children (age 6–17 years) in the GBD super regions

Global burden of disease super region	Prevalence of vision impairment (%)
Central Europe, Eastern Europe & Central Asia	1.9^*^ [51] –17^*^ [44]
High Income	1.2^*^ [52] –5.2^^^ [53]
Latin America & Caribbean	5.8^†^ [54] –12.8^*^ [55]
North Africa & Middle East	2.7^*^ [56] –8.9^¶^ [57]
South Asia	1.49^*^ [58] –1.7^*^ [59]
South-East Asian, East Asia & Oceania	5.9^*^ [60]
Sub-Saharan Africa	3.4^*^ [61] – 3.93^†^ [62]

^†^Presenting visual acuity < 6/12 in the better eye ^*^Presenting visual acuity ≤ 6/12 in the better eye ^^^Presenting visual acuity < 6/9 in the better eye ^¶^Presenting visual acuity ≤ 6/9 in the better eye

### Step 2: Estimate children with eye conditions requiring referral

The estimated number of children requiring services across GBD super regions for both VI and non-vision impairing eye conditions ranged from 3.9–18.9% (Table 2).

**Table 2:** Estimated percentage of children (age 6–17 years) requiring services for eye conditions

Global burden of disease super region	South-East Asia, East Asia & Oceania^*^ (%)	Sub-Saharan Africa^^^ (%)
Vision impairment (total)	17.0	2.0
*Vision impairment due to uncorrected refractive error*	16.5	0.9
Non-vision impairing eye conditions	1.9	1.9
**Total**	**18.9**	**3.9**

^*^^Estimates derived from data contained in the publication by He et al. [44] and Rono et al. [45]

^^^Data reported was limited to vision impairment due to uncorrected refractive error. An estimate of non-vision impairing eye conditions needing services in Sub-Saharan Africa was based on the He et al. data [44]

### Step 3: Cluster survey design

The recommended cluster size for SEHRA is 100 children. This was a pragmatic choice based on the number of examinations a survey team could feasibly complete in one day. A very large school might be selected from the sampling frame more than once, while small schools may not have enough pupils enrolled to complete one cluster. Given a large cluster size of 100, if fewer schools are visited, the precision of the estimate will be lower and will need to be considered during planning.

### Step 4: Estimation of a design effect

The design effect estimated in the SEHRA survey methodology was derived from a literature search of design effects applied to a range of cluster sizes (Supplementary table 1; 2, Additional file 1). Following the sensitivity analysis, a conservative DEFF estimate of 2.0 was chosen for a cluster size of 100 (Supplementary table 3, Additional File 1).

### Step 5: Sample size calculations for different GBD super regions

The minimum and maximum sample size required in a cluster survey to identify school children requiring eye care services (all eye conditions needing services) is 998 and 5,675, represented by the GBD regions South-East Asia, East Asia & Oceania (minimum), and Sub-Saharan Africa (maximum) (Table 3).

**Table 3:** Sample size required for surveys in South-East Asia, East Asia & Oceania and Sub-Saharan Africa

**Global burden of disease super region**	**South-East Asia, East Asia & Oceania**^**^**^	**Sub-Saharan Africa**^†^
All eye conditions needing services	988	5675^*^
All causes of vision impairment needing services	1126	10,777^*^

^^†*^Estimates derived from data contained in a publication by He et al.[44] and Rono et al.[45]

^*^Data reported was limited to vision impairment due to uncorrected refractive error. An estimate of non-vision impairing eye conditions needing services in Sub-Saharan Africa was based on the He et al. data [44]

## Discussion

The reporting of eye health surveys of school children in the literature is partial and inconsistent which may impact the reliability of sample size estimates. In addition, there are only limited published prevalence data to calculate the number of children requiring services in any given location. Several references included in the review were published over 10 years ago, due to a lack of recent studies. This survey uses a modifiable approach as opposed to a standardised methodology, allowing the tool to accommodate to local needs and resources. However, a modifiable tool is limited in the extent to which the data collected are comparable.

The wide range in the prevalence of eye conditions requiring services (VI and non-vision impairing eye conditions) (3.9%-18.9%) means that the sample sizes required to give a precise estimate of the prevalence also vary considerably (from 5,675 to 988). A limitation of this survey design, which has sample sizes large enough to give precise estimates of the overall eye health needs of children, is that estimates of a particular eye condition, such as myopia, will have wider confidence intervals. This means that these sample sizes are not recommended for epidemiological studies of specific eye diseases. It is also important to bear in mind that in settings where the prevalence of eye conditions is low, a rapid assessment of the eye health needs of children may not be feasible given the large sample size required, or the confidence intervals surrounding the estimates will be wide, making it difficult to use the data for planning.

This methodology has several limitations; first, the prevalence estimates for GBD regions were not disaggregated by primary and secondary school-age children due to the scarcity of studies available to represent the highest and lowest prevalence of vision impairment. Further, confidence intervals for prevalence data and the number of children needing services were not calculated. When calculating the estimate of children with non-vision impairing eye conditions, there were insufficient data from each GBD region to calculate the estimate for each region, or to estimate differences between rural and urban areas. Lastly, the precision of estimates may be limited by the number of schools that can be sampled for the cluster size of 100. In regions where fewer schools can be recruited for the survey for the given level of precision, the estimate of the number of children requiring services may not reach the desired level of accuracy and should be considered when using the data for planning.

In future iterations, the SEHRA tool will include functionality to examine teachers. The role of teachers is pivotal in health education and promotion in the school setting [63]. Teachers have an important role in school eye health programmes, including fostering positive perceptions surrounding spectacle wear in children [64, 65]. Involving teachers in SEH programmes, such as providing encouragement, and positive modelling behaviours surrounding spectacle wear has been demonstrated to increase adherence to spectacle wear in school children [66, 67]. Spectacle adherence has also been associated with improved educational performance [68–72].

## Conclusion

In summary, the SEHRA tool will provide a solution to deliver comprehensive school eye health services to children. The SEHRA survey module ‘Assessing the magnitude and nature of local needs’ will equip SEH programme planners with a practical and efficient data collection tool that will provide critical information regarding the magnitude of eye health needs in the target population. The survey will provide useful data for SEH programme planners and implementers, making them better equipped to estimate the resources required to deliver a SEH program, and to decide upon key indicators for use in monitoring the delivery and impact of SEH programmes, including spectacle adherence. The application of this tool will support evidence-based SEH programme design, planning, monitoring and evaluation, and will be of value to other SEH stakeholders, including funders, researchers, and policymakers.

## Supplementary Information

**Additional file 1: Table 1.** Cluster size and design effect reported in school-based cluster surveys for non-eye health related conditions.** Table 2.** Cluster size and design effects reported in school-based eye health surveys.** Table 3.** Modelling the effect of changing the design effect on the sample size.

## Data Availability

Not applicable.

## References

[1] Olitsky SE, Nelson BA, Brooks S (2002). The sensitive period of visual development in humans. J Pediatr Ophthalmol Strabismus.

[2] Dirani M, Zhang X, Goh LK, Young TL, Lee P, Saw SM (2010). The role of vision in academic school performance. Ophthalmic Epidemiol.

[3] Chadha RK, Subramanian A (2011). The effect of visual impairment on quality of life of children aged 3–16 years. Br J Ophthalmol.

[4] Schneider J, Leeder SR, Gopinath B, Wang JJ, Mitchell P (2010). Frequency, course, and impact of correctable visual impairment (uncorrected refractive error). Surv Ophthalmol.

[5] Rainey L, Elsman EBM, van Nispen RMA, van Leeuwen LM, van Rens G (2016). Comprehending the impact of low vision on the lives of children and adolescents: a qualitative approach. Qual Life Res.

[6] Burton M, Ramke J, Marques A, Bourne R, Congdon N, Jones I, et al. The Lancet Global Health Commission on Global Eye Health: vision beyond 2020. Lancet Glob Health. 2021;9:489–551.

[7] Zhao J, Pan X, Sui R, Munoz SR, Sperduto RD, Ellwein LB (2000). Refractive error study in children: results from Shunyi District. China Am J Ophthalmol.

[8] Maul E, Barroso S, Munoz S, Sperduto R, Ellwein L (2000). Refractive error study in children: results from La Florida. Chile Am J Ophthalmol.

[9] Pokharel GP, Negrel AD, Munoz SR, Ellwein LB (2000). Refractive error study in children: results from Mechi Zone. Nepal Am J Ophthalmol.

[10] Huang J, Wen D, Wang Q, McAlinden C, Flitcroft I, Chen H, Saw SM, Chen H, Bao F, Zhao Y (2016). Efficacy comparison of 16 interventions for myopia control in children: a network meta-analysis. Ophthalmology.

[11] Seet B, Wong TY, Tan DT, Saw SM, Balakrishnan V, Lee LK, Lim AS (2001). Myopia in Singapore: taking a public health approach. Br J Ophthalmol.

[12] Flitcroft DI (2012). The complex interactions of retinal, optical and environmental factors in myopia aetiology. Prog Retin Eye Res.

[13] Ikuno Y (2017). Overview of the complications of high myopia. Retina.

[14] Gordon RA, Donzis PB (1985). Refractive development of the human eye. Arch Ophthalmol.

[15] Pascual M, Huang J, Maguire MG, Kulp MT, Quinn GE, Ciner E, Cyert LA, Orel-Bixler D, Moore B, Ying G-S (2014). Risk factors for amblyopia in the vision in preschoolers study. Ophthalmology.

[16] Kulp MT, Ciner E, Maguire M, Moore B, Pentimonti J, Pistilli M, et al. Uncorrected Hyperopia and Preschool Early Literacy: Results of the Vision in Preschoolers–Hyperopia in Preschoolers (VIP-HIP) Study. Ophthalmol. 2016;123(4):681–89. 10.1016/j.ophtha.2015.11.023 PMC4808323 26826748

[17] Ip JM, Robaei D, Kifley A, Wang JJ, Rose KA, Mitchell P (2008). Prevalence of hyperopia and associations with eye findings in 6- and 12-year-olds. Ophthalmology.

[18] Ciner EB, Kulp MT, Pistilli M, Ying GS, Maguire M, Candy TR, et al. Vision In Preschoolers - Hyperopia In Preschoolers (VIP-HIP) Study Group†. Associations between visual function and magnitude of refractive error for emmetropic to moderately hyperopic 4- and 5-year-old children in the Vision in Preschoolers - Hyperopia in Preschoolers Study. Ophthalmic Physiol Opt. 2021;41(3):553–64. 10.1111/opo.12810 PMC10749558 33772848

[19] Kulp M, Ying G-S, Huang J, Maguire M, Quinn G, Ciner E, et al. Associations between Hyperopia and Other Vision and Refractive Error Characteristics. Optom Vis Sci. 2014;91:383–9. 10.1097/OPX.0000000000000223 PMC4051821 24637486

[20] Group V-HS, Kulp MT, Ciner E, Maguire M, Moore B, Pentimonti J, Pistilli M, Cyert L, Candy TR, Quinn G (2016). Uncorrected hyperopia and preschool early literacy: results of the Vision in Preschoolers-Hyperopia in Preschoolers (VIP-HIP) study. Ophthalmology.

[21] Fulton AB, Hansen RM, Petersen RA (1982). The relation of myopia and astigmatism in developing eyes. Ophthalmology.

[22] Sjöstrand J, Abrahamsson M. Risk factors in amblyopia. Eye. 1990;4:787–93. 10.1038/eye.1990.12445. 10.1038/eye.1990.124 2101108

[23] Gwiazda J, Grice K, Held R, McLellan J, Thorn F (2000). Astigmatism and the development of myopia in children. Vision Res.

[24] Singh V, Malik KPS, Malik VK, Jain K (2017). Prevalence of ocular morbidity in school going children in West Uttar Pradesh. Indian J Ophthalmol.

[25] Kusunoki T, Morimoto T, Nishikomori R, Yasumi T, Heike T, Fujii T, Nakahata T (2009). Changing prevalence and severity of childhood allergic diseases in Kyoto, Japan, from 1996 to 2006. Allergol Int.

[26] Ben Kumah D, Lartey S, Yemanyi F, Boateng E, Awuah E (2015). Prevalence of allergic conjunctivitis among basic school children in the Kumasi Metropolis (Ghana): A community-based cross-sectional study. BMC Ophthalmol.

[27] Nishima S (2013). A study on the prevalence of allergic diseases in school children in western districts of Japan: comparison between the studies in 1992 and 2002 with the same methods and same districts. Nihon Shoni Arerugi Gakkaishi Japanese J Pediatric Allergy Clinical Immunology.

[28] McCormick A, Fleming D, Charlton J, Britain G (1995). Surveys OoPCa, Britain G, Security DoH, social: morbidity statistics from general practice : fourth national study 1991–1992.

[29] Rose P, Harnden A, Brueggemann A, Perera R, Sheikh A, Crook D, Mant D (2005). Chloramphenicol treatment for acute infective conjunctivitis in children in primary care: a randomised double-blind placebo-controlled trial. Lancet.

[30] Rao GN, Sabnam S, Pal S, Rizwan H, Thakur B, Pal A (2018). Prevalence of ocular morbidity among children aged 17 years or younger in the eastern India. Clin Ophthalmol.

[31] Kumar R, Mehra M, Dabas P (2004). Kamlesh, Raha R: a study of ocular infections amongst primary school children in Delhi. J Commun Dis.

[32] Puri S, Bhattarai D, Adhikari P, Shrestha JB, Paudel N (2015). Burden of ocular and visual disorders among pupils in special schools in Nepal. Arch Dis Child.

[33] Shaikh SP, Aziz TM (2005). Pattern of eye diseases in children of 5–15 years at Bazzertaline Area (South Karachi) Pakistan. JCPSP.

[34] Nepal BP, Koirala S, Adhikary S, Sharma AK (2003). Ocular morbidity in schoolchildren in Kathmandu. Br J Ophthalmol.

[35] Zhang S-y, Li J, Liu R, Lao HY, Fan Z, Jin L, et al. Association of Allergic Conjunctivitis With Health-Related Quality of Life in Children and Their Parents. JAMA Ophthalmology. 2021;139(8):830–7. 10.1001/jamaophthalmol.2021.1708 PMC8193548 34110380

[36] Vichyanond P, Pacharn P, Pleyer U, Leonardi A (2014). Vernal keratoconjunctivitis: a severe allergic eye disease with remodeling changes. Pediatr Allergy Immunol.

[37] Education - Every Child Has The Right To Learn [https://www.unicef.org/education]

[38] Simons HD, Gassler PA (1988). Vision anomalies and reading skill: a meta-analysis of the literature. Am J Optom Physiol Opt.

[39] Williams WR, Latif AHA, Hannington L, Watkins DR (2005). Hyperopia and educational attainment in a primary school cohort. Arch Dis Child.

[40] Ciner EB, Dobson V, Schmidt PP, Allen D, Cyert L, Maguire M, Moore B, Orel-Bixler D, Schultz J (1999). A survey of vision screening policy of preschool children in the United States. Surv Ophthalmol.

[41] Holguin AMC, Congdon N, Patel N, Ratcliffe A, Esteso P, Flores ST, Gilbert D, Rito MAP, Munoz B (2006). Factors associated with spectacle-wear compliance in school-aged Mexican children. Invest Opthalmol Vis Sci.

[42] Braun V, Clarke V (2006). Using thematic analysis in psychology. Qual Res Psychol.

[43] Murray CJL, Ezzati M, Flaxman AD, Lim S, Lozano R, Michaud C, Naghavi M, Salomon JA, Shibuya K, Vos T (2012). GBD 2010: design, definitions, and metrics. The Lancet.

[44] He M, Huang W, Zheng Y, Huang L, Ellwein LB (2007). Refractive error and visual impairment in school children in Rural Southern China. Ophthalmology.

[45] Karimurio J: The “Up-Scaling Screening of Children Using Children Portable Eye Examination Kit (Peek) in Schools in Trans Nzoia County, Kenya". In.: University of Nairobi; 2019:35.

[46] Kuper H, Polack S, Limburg H (2006). Rapid assessment of avoidable blindness. Community eye health.

[47] International classification of diseases for mortality and morbidity statistics (11th Revision) [https://icd.who.int/browse11/l-m/en]

[48] Casson RJ, Kahawita S, Kong A, Muecke J, Sisaleumsak S, Visonnavong V (2012). Exceptionally low prevalence of refractive error and visual impairment in schoolchildren from Lao People's Democratic Republic. Ophthalmology.

[49] Paudel P, Ramson P, Naduvilath T, Wilson D, Phuong HT, Ho SM, Giap NV (2014). Prevalence of vision impairment and refractive error in school children in Ba Ria - Vung Tau province. Vietnam Clin Exp Ophthalmol.

[50] Ezinne N, Mashige K: Refractive error and visual impairment in primary school children in Onitsha, Anambra State, Nigeria. African Vision and Eye Health 2018;77:98–105.

[51] Gao Z, Meng N, Muecke J, Chan WO, Piseth H, Kong A, Jnguyenphamhh T, Dehghan Y, Selva D, Casson R (2012). Refractive error in school children in an urban and rural setting in Cambodia. Ophthalmic Epidemiol.

[52] Naidoo KS, Raghunandan A, Mashige KP, Govender P, Holden BA, Pokharel GP, Ellwein LB (2003). Refractive error and visual impairment in African children in South Africa. Invest Ophthalmol Vis Sci.

[53] Megbelayin E (2013). Visual impairment among school children -calabar vision screening survey in secondary Schools (CVS4 Study). Int J Ophthalmolog Vis Sci.

[54] Saxena R, Vashist P, Tandon R, Pandey RM, Bhardawaj A, Menon V, Mani K (2015). Prevalence of myopia and its risk factors in urban school children in Delhi: the North India Myopia Study (NIM Study). PLoS One.

[55] Sharma IP, Lepcha NT, Lhamo T, Ellwein LB, Pokharel GP, Das T, Sapkota YD, Dorji T, Peldon S (2020). Visual impairment and refractive error in school children in Bhutan: The findings from the Bhutan School Sight Survey (BSSS 2019). PLoS One.

[56] Salomão SR, Cinoto RW, Berezovsky A, Mendieta L, Nakanami CR, Lipener C, Muñoz EdH, Ejzenbaum F, Belfort R, Pokharel GP (2008). Prevalence and causes of visual impairment in low-middle income school children in Sao Paulo. Brazil. Invest Ophthalmol Vis Sci..

[57] Sauer T, Martín M, Alarcón J, Zunt J (2016). Prevalence of vision impairment in school children of Puente Piedra, Peru. Vis Pan-Am Pan-Am J Ophthalmolog.

[58] Yekta A, Fotouhi A, Hashemi H, Dehghani C, Ostadimoghaddam H, Heravian J, Derakhshan A, Yekta R, Behnia M, Khabazkhoob M (2010). Prevalence of refractive errors among schoolchildren in Shiraz. Iran Clin Exp Ophthalmol.

[59] Fotouhi A, Hashemi H, Khabazkhoob M, Mohammad K (2007). The prevalence of refractive errors among schoolchildren in Dezful Iran. Br J Ophthalmol.

[60] Popović-Beganović A, Zvorničanin J, Vrbljanac V, Zvorničanin E (2018). The prevalence of refractive errors and visual impairment among school children in Brčko District Bosnia and Herzegovina. Semin Ophthalmol.

[61] Harrington SC, Stack J, Saunders K, O'Dwyer V (2019). Refractive error and visual impairment in Ireland schoolchildren. Br J Ophthalmol.

[62] Rose K, Younan C, Morgan I, Mitchell P (2003). Prevalence of undetected ocular conditions in a pilot sample of school children. Clin Exp Ophthalmol.

[63] Jourdan D, Samdal O, Diagne F, Carvalho GS (2008). The future of health promotion in schools goes through the strengthening of teacher training at a global level. Promot Educ.

[64] Thummalapalli R, Williams JD, Khoshnood K, Salchow DJ, Forster SH (2012). Effect of education sessions of a structured school eye screening programme on Indian schoolteachers’ knowledge and responsibility for children’s eye health. Health Educ J.

[65] Burnett AM, Yashadhana A, Lee L, Serova N, Brain D, Naidoo K (2018). Interventions to improve school-based eye-care services in low- and middle-income countries: a systematic review. Bull World Health Organ.

[66] Morjaria P, Evans J, Gilbert C (2019). Predictors of spectacle wear and reasons for nonwear in students randomized to ready-made or custom-made spectacles: results of secondary objectives from a randomized noninferiority trial. JAMA Ophthalmology.

[67] Yi H, Zhang H, Ma X, Zhang L, Wang X, Jin L, Naidoo K, Minto H, Zou H, Lu L (2015). Impact of free glasses and a teacher incentive on children's use of Eyeglasses: a cluster-randomized controlled trial. Am J Ophthalmol.

[68] Ma Y, Congdon N, Shi Y, Hogg R, Medina A, Boswell M, Rozelle S, Iyer M (2018). Effect of a local vision care center on eyeglasses use and school performance in Rural China: a cluster randomized clinical trial. JAMA Ophthalmology.

[69] Ma X, Zhou Z, Yi H, Pang X, Shi Y, Chen Q, Meltzer ME, le Cessie S, He M, Rozelle S (2014). Effect of providing free glasses on children’s educational outcomes in China: cluster randomized controlled trial. BMJ : British Medical Journal.

[70] Hannum E, Zhang Y (2012). Poverty and proximate barriers to learning: vision deficiencies, vision correction and educational outcomes in Rural Northwest China. World Dev.

[71] Glewwe P, Park A, Zhao M (2016). A better vision for development: eyeglasses and academic performance in rural primary schools in China. J Dev Econ.

[72] Glewwe P, West KL, Lee J (2018). The impact of providing vision screening and free eyeglasses on academic outcomes: evidence from a Randomized Trial in title i elementary schools in Florida. J Policy Anal Manage.

