# Supplementary material for: A School Eye Health Rapid Assessment (SEHRA) planning tool: Module to survey the magnitude and nature of local needs
Source: BMC Public Health. 2022 Sep 2;22:1665. doi: 10.1186/s12889-022-13927-x (PMC9437397; doi:10.1186/s12889-022-13927-x)
Supplement: Supplementary file 1 — Additional file 1: Table 1. Cluster size and design effect reported in school-based cluster surveys for non-eye health related conditions. Table 2. Cluster size and design effects reported in school-based eye health surveys. Table 3. Modelling the effect of changing the design effect on the sample size. [file 12889_2022_13927_MOESM1_ESM.docx]

**Table 1. Cluster size and design effect reported in school-based cluster surveys for non-eye health** **related conditions**

| **Author** | **Study design** | **Area of interest** | **Cluster size** | **Design effect** |
| --- | --- | --- | --- | --- |
| Kim et al.^[1]^ | Cluster randomised control trial | Enterobiasis | 21 | 3.0 |
| De Bock et al.^[2]^ | Cluster randomised control trial | Physical activity | 9–46 | 3.0 |
| Bunten et al.^[3]^ | Cluster randomised control trial | Health behaviour | 106 | 5.7 |
| Majstorov et al.^[4]^ | School-based survey | Thyroid volume | 40 | - |
| Taiaroa et al.^[5]^ | School-based survey | Scabies and impetigo | 2–9 | 1.0 |
| Wahdana et al.^[6]^ | School-based survey | Injury | 40 | 1.4 |
| Ahmed et al.^[7]^ | School-based survey | Food addiction | 40 | 1.4 |
| Shyam et al.^[8]^ | School-based survey | Dental fluorosis | 40 | 1.9 |
| Mahanta et al.^[9]^ | School-based survey | Obesity | 50 | 2.0 |
| Naotunna et al.^[10]^ | School-based survey | Nutritional status | 50 | 2.0 |
| Gibbs et al.^[11]^ | School-based survey | Impact of school-based programme | 40 | 3.0 |
| Kumar et al.^[12]^ | School-based survey | Exercise and eating habits | 75 | 13.8 |

**Table 2. Cluster size and design effects reported in school-based eye health surveys**

| **Author** | **Prevalence (%)*** | **Design Effect** | **Cluster size** | **Additional Information** |
| --- | --- | --- | --- | --- |
| Casson et al.^[13]^ | 16 | 1.25 | NA | Prevalence of uncorrected refractive error |
| Ezinne et al.^[14]^ | 15 | NA | 102 | Prevalence of all causes of vision impairment |
| Paudel et al.^[15]^ | 25 | 1.50 | 40 | Prevalence of uncorrected refractive error |
| He et al.^[16]^ | 25 | 2.00 | 50-80 | Prevalence of all causes of vision impairment |
| Salomão et al.^[17]^ | 20 | 0.54–1.71 | 215–430 | Prevalence of all causes of vision impairment |

*Estimated prevalence used to calculate sample size

**Table 3. Modelling the effect of changing the design effect on the sample size**

| **Global burden of disease super Region** | **Prevalence (%)*** | **Design effect** | **Sample size^** |
| --- | --- | --- | --- |
| South-East Asia, East Asia & Oceania | 18.9 | 1.25 | 618 |
|  |  | 1.50 | 741 |
|  |  | 2.00 | 988 |
|  |  | 3.00 | 1544 |
| Sub-Saharan Africa | 3.9 | 1.25 | 3547 |
|  |  | 1.50 | 4256 |
|  |  | 2.00 | 5675 |
|  |  | 3.00 | 8513 |

^ Based on a cluster size of 100 students *All referable eye conditions

**Supplementary information**

The ‘Standard Guidelines for Comprehensive School Eye Health Programs’ recommends a VA cutoff of ≤6/9.[18] If a threshold of <6/9 is used, there will be an increase in the prevalence, thereby reducing the required sample size required. This will increase the rate of false positives or inconclusive results from screening, leading to wasted programme resources, wasted beneficiary time and resources. The SEHRA tool will include an optional additional function to measure VA at the 6/9 threshold, maintaining VA measurement at 6/12 to allow international comparison of the eREC coverage indicator.

**References**

1. Kim DH, Yu HS: **Effect of a one-off educational session about enterobiasis on knowledge, preventative practices, and infection rates among schoolchildren in South Korea**. *PLoS One* 2014, **9**(11):e112149-e112149.

2. De Bock F, Genser B, Raat H, Fischer JE, Renz-Polster H: **A Participatory Physical Activity Intervention in Preschools: A Cluster Randomized Controlled Trial**. *Am J Prev Med* 2013, **45**(1):64-74.

3. Bunten A, Porter L, Burgess-Allen J, Howell-Jones R, Jackson J, Ward D, Staples V, Staples P, Rowthorn H, Saei A *et al*: **Using behavioural insights to reduce sugar in primary school children's packed lunches in derby; A cluster randomised controlled trial**. *Appetite* 2021, **157**:104987-104987.

4. Majstorov V, Miladinova D, Kuzmanovska S, Ittermann T, Pop Gjorcheva D, Vaskova O, Ugrinska A, Milevska Kostova N, Karanfilski B: **Schoolchildren thyroid volume in North Macedonia: data from a national survey in an iodine-sufficient country**. *Journal of Endocrinological Investigation* 2020, **43**(8):1073-1079.

5. Taiaroa G, Matalavea B, Tafuna'i M, Lacey J, Price D, Isaia L, Leaupepe H, Viali S, Lee DYJ, Gorrie C *et al*: **Scabies and impetigo in Samoa: A school-based clinical and molecular epidemiological study**. *The Lancet Regional Health: Western Pacific* 2021, **6**.

6. Wahdan MM, Sayed AM, Abd Elaziz KM, El-Hoseiny MM, Al-Gwaily MM: **Prevalence of injuries among high school students in Eastern and Western parts of Cairo, Egypt**. *Injury* 2016, **47**(12):2650-2654.

7. Ahmed AY, Sayed AM: **Prevalence of food addiction and its relationship to body mass index**. *Egyptian Journal of Medical Human Genetics* 2017, **18**(3):257-260.

8. Shyam R, Bhadravathi Chaluvaiah M, Kumar A, Pahwa M, Rani G, Phogat R: **Impact of dental fluorosis on the oral health related quality of life among 11- to 14-year-old school children in endemic fluoride areas of Haryana (India)**. *Int Dent J* 2020, **70**(5):340-346.

9. Mahanta T, Mahanta B, Baruah S, Deuri A, Rasailey R: **Prevalence and determinant of obesity amongst school going adolescent of Assam**. *J Evid Based Med Healthc* 2017, **4**:2349-2562.

10. Naotunna NP, Dayarathna M, Maheshi H, Amarasinghe GS, Kithmini VS, Rathnayaka M, Premachandra L, Premarathna N, Rajasinghe PC, Wijewardana G *et al*: **Nutritional status among primary school children in rural Sri Lanka; a public health challenge for a country with high child health standards**. *BMC Public Health* 2017, **17**(1):57-57.

11. Gibbs L, Staiger PK, Johnson B, Block K, Macfarlane S, Gold L, Kulas J, Townsend M, Long C, Ukoumunne O: **Expanding children's food experiences: the impact of a school-based kitchen garden program**. *J Nutr Educ Behav* 2013, **45**(2):137-146.

12. Kumar S, Ray S, Roy D, Ganguly K, Dutta S, Mahapatra T, Mahapatra S, Gupta K, Chakraborty K, Das MK *et al*: **Exercise and eating habits among urban adolescents: a cross-sectional study in Kolkata, India**. *BMC Public Health* 2017, **17**(1):468-468.

13. Casson RJ, Kahawita S, Kong A, Muecke J, Sisaleumsak S, Visonnavong V: **Exceptionally low prevalence of refractive error and visual impairment in schoolchildren from Lao People's Democratic Republic**. *Ophthalmology* 2012, **119**(10):2021-2027.

14. Ezinne N, Mashige K: **Refractive error and visual impairment in primary school children in Onitsha, Anambra State, Nigeria**. *African Vision and Eye Health* 2018, **77**.

15. Paudel P, Ramson P, Naduvilath T, Wilson D, Phuong HT, Ho SM, Giap NV: **Prevalence of vision impairment and refractive error in school children in Ba Ria - Vung Tau province, Vietnam**. *Clin Exp Ophthalmol* 2014, **42**(3):217-226.

16. He M, Huang W, Zheng Y, Huang L, Ellwein LB: **Refractive Error and Visual Impairment in School Children in Rural Southern China**. *Ophthalmology* 2007, **114**(2):374-382.

17. Salomão SR, Cinoto RW, Berezovsky A, Mendieta L, Nakanami CR, Lipener C, Muñoz Ede H, Ejzenbaum F, Belfort R, Jr., Pokharel GP *et al*: **Prevalence and causes of visual impairment in low-middle income school children in Sao Paulo, Brazil**. *Invest Ophthalmol Vis Sci* 2008, **49**(10):4308-4313.

18. Gilbert C, Minto H, Morjaria P, Khan I: **Standard Guidelines for Comprehensive School Eye Health Programs**. In*.* London: London School of Hygiene and Tropical Medicine; 2016.
